# Human Parasites

**Published:** 1883-12

**Authors:** F. W. Goding

**Affiliations:** President of the Linnæan Science Club, Ancona, Ill.


### ARTICLE II.

HUMAN PARASITES. By F. W. GODING, M.D., President of the Linnæan Science Club, Ancona, Ill.

### INTRODUCTION.

A parasite is, according to Webster, "an animal which lives during the whole or a part of its existence on the body of some other animal."*

Van Beneden † defines it thus: "The parasite is he whose profession it is to live at the expense of his neighbor and whose

*From *parasitus*, to eat at the table of another.

†Animal parasites and messmates.

only employment consists in taking advantage of him, but prudently, so as not to endanger his life. He is a pauper who needs help, lest he should die on the public highway, but who practices the precept not to kill the fowl in order to get the eggs." Parasites belong to numerous different types ranging through the entire animal kingdom and the dissimilarity should be understood to get a correct idea of the subject.

Among mammals, birds, reptiles, and amphibians we do not find any exclusively parasitic types, but in each are some which to a certain extent are. Thus, the vampire bats live upon the blood of other mammals; the bald eagle depends to a considerable extent on the fish-hawk for food. There are a few fishes which are partly parasitic, e.g., some of the *Fieras feridæ* inhabit the intestines of holothurians and other echinoderms; the *stegophilus* lives in the mouth of another cat-fish.* Many of the invertebrates are parasites, *e. g.*, the *entoconcha* lives in the intestines of echinoderms; the *cochiolepis parasiticus* is found under the scales of a worm.

Among insects, the true coleopterous types, *meloidæ*, *pselaphidæ* and *staphylinidæ* are nearly all parasitic. Of the *arachnoids* we have the Linguatulidæ and Acaridæ† infesting the skin and air passages of man.

The Crustaceans present a number of types among which may be mentioned the *Pinnotheres*, *conchodytes*, etc. Of the true worms are the leeches. Of the rotifers the *Bellatro calvus* is found in certain worms.

Among scolices, we have animals which live almost altogether in other animals, as Cestodes, Trematodes, Nematodes, etc., which will be more fully described further on.

The Polyps which are parasitic are few in number; among others are *Epizoanthus americanus* attached to the hermit crab‡ and *Polythoa* which infests a peculiar form of sponge (*Hyalonema*) found off the coasts of China and southwestern Europe.

The parasitic species contributed by the Protozoans are worm-like forms, known as Gregarinæ, inhabiting the intestines of

**Platysloma.*

†See chart.

‡*Eupagurus pubescens.*

vertebrates and invertebrates, especially abundant in the intestinal canal of beetles, cock-roaches, etc. Hence, it will be seen that parasites vary greatly in type, having nothing in common except the physiological character of depending upon others for sustenance.

Scarcely any portion of the human body is free from parasites; and each organ and system has its own special parasites.

Davaine* has accordingly divided them into four groups, viz.: (1) those which inhabit the cavities communicating with the exterior, *e. g.*, (*a*) the respiratory passages; (*b*) the digestive passages; (*c*) the biliary passages, and (*d*) the urinary passages. (2) Those which are contained in closed cavities, natural or accidental, as, (*a*) blood-vessels; (*b*) serous cavities. (3) Those which belong to some organic system, (*a*) the nervous system; (*b*) the muscles of animal life; (*c*) the lymphatic system, and (*d*) the inter-organic cellular tissue. (4) Those which affect complex organs, as (*a*) eye; (*b*) the genital organs. But to treat the subject scientifically it is better to study them with reference to their natural history.†

The study of parasites has received considerable attention from European writers, where their presence is much more common than here, but they have been sufficiently often met with here, and with a fatal result, that a more extended study of them is imperatively demanded.

How parasites were developed in the interior of the body was, until a comparatively recent period, a sealed book; but through the earnest investigations of Bremser, Le Clerc, C. Davaine, Cobbold, Diesing, Dujardin, and others, we are now able to study them with facility tracing them to the ovum, and conversely from the ovum to the mature animal. One peculiarity is that they rarely pass through more than one stage while in the interior of a certain animal, waiting for its next host to aid in its progress.

PART I.

ENTOZOA.—Parasites are divided into entozoa and ectozoa‡.

*Davaine, C. Traite des Entozoaires, etc., Paris, 1879.

†See the chart.

‡Epizoa of some writers.

The former are further subdivided into *arachnoides*, *platyhelminthes*, *Nematelminthes*, and *Infusoria*.

*Arachnoides* are the spider-like* parasites. The only order which is found within the body is Linguatulida, consisting of but one family, Pentastomidæ, belonging to which are two individuals, viz.:

*P. denticulatum.*—This is the larval form of the *P. tænoides*, in which state it, as a rule, exists in man, though one case has been reported where the perfect animal was dislodged from the nose†. This parasite is found quite frequently in human bodies, encysted in the liver. The adult, *P. tænoides*, has a lancet shaped body, flattened over the abdomen, smallest at the tail, and marked with 90 transverse rings. The cephalo-thoracic segments are continuous with the body, and on each are a pair of retractile claws. The head has an oval mouth, armed with a horny lip. Breathing pores are numerous in all parts of the body, excepting the cephalic segment.

The length of the adult male is about an inch, while the female measures three or four inches.

The genital aperture of the former is found at the front part of the abdomen, while in the female it is placed at the caudal extremity. This parasite is oviporous, and passes through a complete metamorphosis, the first stage being that of the embryo, with a boring apparatus; the second, a motionless pupa; the third, or larval, having numerous rows of spines and two pairs of double claws; the fourth, the perfect state. It is while in the third stage that it is called *P. denticulatum*, and is of most interest to us. (Length, 2½ lines).

The germs are usually introduced into the human stomach with raw fruits and vegetables, on which they are thrown by the host from its nose when sneezing.

Hence, we should be watchful when fondling dogs, as they are most liable to the trouble. Also, all fruits and vegetables should be carefully cleansed before being eaten.

As the parasite gives rise to no functional derangement, it is unnecessary to treat of it clinically.

---

* Mites, ticks, etc.

† Dr. Landon, of Elbing, *Berl. Klin.*, *Woch.* No. 49, 1878.

*P. constrictum.*—The natural history of this parasite is as yet incomplete (so far as is known), the larval form only having been described. It is possible that the mature animal has been described under some other name. However harmless the *P. denticulatum* may be, this parasite is to be dreaded, as it has been known to cause peritonitis and pneumonitis, and death in at least two cases.

The length of *P. constrictum* is nearly three-quarters of an inch; width, one-tenth of an inch. The cephalo-thorax is provided with four foot claws, and the abdomen is made up of twenty-three rings.

They are found encysted in the liver and lungs, coiled up like a watch spring in small sacs throughout the whole organ.

"When cut into, worms were seen regularly encysted in its substance, appearing as yellow specks."

*Treatment.*—As it would be impossible to diagnosticate this parasite, the principle to follow would be to meet the indications as they arise.

PLATYHELMINTHES.*—This class is divided into two orders, viz.: CESTODES and TREMATODES. CESTODES† are ribbon-like animals, some barely visible, others 100 feet long, living while in the larval state in the tissues, and as mature parasites in the intestines. They are divided into bi-sexual segments growing from the neck, and are discharged from the bowels, living a sort of individual existence. They have neither mouth nor organs of digestion, the animal to which they are attached performing the digestive act for them, the food being absorbed through the integument.

The segments impregnate themselves, become detached, are passed from the intestine, and swelling up they burst, the ova being scattered by the winds and water over the grasses, etc. The grass is then carried to the stomach of some animal, the ovum hatched into an embryo, which pierces the walls of that organ, entering the blood vessels, and finally works its way into its appropriate tissue. It now becomes encysted, and forms the so-called "hydatid." If the tissue is eaten without being properly cooked, the cysts become developed into some form of tape-

---

* Broad worms.

† Cestura bands.

worm, being freed by the solvent power of the gastric juice.

The order of cestodes is divided into two families, viz., TÆNIDÆ, which have hooks and suckers on the head, and the genital organs located on the lateral edge of each segment.

BOTHRIOCEPHALIDÆ.—*T. solium** is of a tape-like form, composed of a series of bodies, each containing male and female sexual organs, and capable of self-impregnation. It resides in the intestine.

This parasite exists in two forms within the human body, viz., cystic,† in the tissue; and the sexually perfect worm, in the intestines.

It is from 15 to 25 feet long, $\frac{1}{4}$ to $\frac{1}{2}$ inch wide, and white. The head is 1-50 of an inch thick, with about 26 hooks, arranged in two series radiating outward. The common genital orifice is located at the center of the lateral border of each articulation.

The eggs are spherical, and 1-750 of an inch in diameter.

As the articulations mature, they pass from the bowel, retaining their vitality for several days. They possess the power of locomotion in a slight degree, thus being enabled to attach themselves to the grass, etc., where they have fallen. Later, they are conveyed into the stomach of some animal, where the eggs are hatched, the embryo remaining free. They rapidly bore through the walls of the intestine, aided by three pairs of spicules with which they are provided, and find their way into the tissues of the body, there to become encysted. They increase in size, and are now in the next stage of development; the six spicules are discarded, the parasite acquires a neck and head, which is provided with four suckers, and a crown of hooks similar to those of the adult tapeworm. The head and neck are inverted upon themselves, and surrounded by a globular vesicle, the body. This is known as "*cysticercus cellulosæ*,"‡ or "hydatid," and when found in the hog, the meat is called "measly pork." The parasite may remain in this state for an unlimited period of time without change, unless it be a gradual atrophy or the cyst after several years.

* Usually found alone.

† Cysticercus cellulosæ.

‡ From its situation.

The subsequent history deals with introduction of the *C. cellulosæ* into the human intestine, and its development into the adult tapeworm.

When a piece of pork containing cysticerci is conveyed into the human stomach in a partly cooked condition, the sac is digested by the gastric juice, liberating the parasite. It attaches itself by means of the hooks and suckers to the upper surface of the intestine, where the neck, slowly absorbing nutriment, increases in length until developed into the perfect tænia.

*Symptoms.*—A great variety of symptoms have been given supposed to be due to the presence of this parasite, but the only one of diagnostic value is the passage of fragments from the bowels. Healthy persons may be infested for years without inconvenience, while those of feeble constitution will suffer from abdominial pains, indigestion, intestinal hæmorrhage, restlessness and even convulsions.

*Treatment.*—The principles to be followed in the treatment of tænia is to first produce a toxic effect upon them as they will then loosen their hold upon the mucous membrane and cause their expulsion from the bowels. Among the multitude of remedies, the following have been found in many cases efficacious: Ol. terebinthinæ, filix mas (ethereal ext.), koosso, kameela, carbolic acid, pomgranate root bark and pumpkin seed tea. The above are all of value, and it may be necessary to try several before one will be found to dislodge the worm. The manner of using the remedy is of as much importance as its selection. A very good plan is to first move the bowels slightly; second, place the patient on a light diet; third, give a teaspoonful of the following prescription after each meal in a tablespoonful of water for three days; then if the worm is not dislodged, fourth, give a slight purge:

| | | | |
|---|---|---|---|
| ℞ | Granati radicis cort. | 90. c. c. or grs | ℥iij |
| | *Ext. filicis fluidum | 15. " " | ℥ss |
| | Syrup rhei | 45. " " | ℥jss |

Mix. Sig., take as directed above.

* Ethereal extract

*Cysticercus cellulosæ.**—This is in very close relation with the above, and it is sometimes found in man. We will devote a few lines to it.

This parasite is found in the following localities: In the cellular tissue beneath the corium, between and in the muscles, on the surface and in the interior of the brain, in the envelopes and inner chambers of the eyeballs, in the heart, lungs, mesentery and lymphatic glands.

*Symptoms.*—The symptoms depend on the number of parasites and the importance of the organ or tissue infested. In the subcutaneous cellular tissue they are usually harmless, while in the eye one may seriously interfere with vision; and in the brain the local irritation may cause vertigo, paralysis, convulsions and death. One case is recorded† in which a young man twenty-two years of age, apparently in good health, fell dead in the street, the the fatal result being due to the presence of a cysticercus within the *pons varolii.* A correct diagnosis was impossible during life. When subcutaneously located they are sometimes felt as small round hard bodies.

*Treatment.*—The treatment is excision of the cyst where practicable, but it may be the plan recommended by Prof. Rand for treating "trichanosis," (q. v.) will be useful.

The parasites are undoubtedly introduced into the system by swallowing food containing the ova, as meats, and more frequently green tops of garden vegetables. Hence such articles of food should be well cleaned and properly cooked.

*C. tenuicollis.*—This is the larva of *tœnia marginata* and evidence proving its presence as a parasite in the human body is slight, but Cobbold considers that two undoubted cases are known. What was stated about the above will be sufficient for this species. The mature worm is common in the dog.

*Tœnia acanthotrias.*—This is identical in appearance with *T. solium*, and is considered by good authorities to be a mal-formed variety of that parasite, the only difference being three rows of hooks on its rostellum, while the *T. solium* has but two.

*T. medio-canellata.*— This cestode is derived from the *cys-*

---

† For an account of its development see Tolman.

‡ Cobbold "Parasites," p. 92.

*ticercus bovis* found in beef. It very closely resembles *T. solium* being, however, much larger and stouter. The head is club-shaped with four suckers and a depression in the center corresponding to the rostellum of its cousin ; but the coronet of hooks is wanting.

This parasite varies from fifteen to twenty feet in length.

*Symptoms.*—See *T. solium.*

*Treatment.*—The expulsion of this parasite is much more easily accomplished than that of *T. solium* the same method being used.

*Echinococcus hominis.*—This parasite with its sac and contained liquid is commonly called "hydatid." It is a juvenile stage of *T. echinococcus* found in the dog and wolf, and it developed into that state upon gaining entrance into the alimentary canal of those animals. Each parasite consists of a body and head. Around the latter is found a row of sharp hook-like teeth. The body is solid and a number of ovoid bodies (ova) beneath the external coat gives it a speckled appearance. The length of the perfect cestode as found in the dog in from one-fourth to one inch.

The *E. hominis* is found in the liver, ovaries, lungs, kidneys, testicles, brain, muscles, bones§, uterus, spleen, heart, etc.

Hydatids are dangerous from pressure upon neighboring organs interfering with their action, or they ulcerate and rupture causing death from contact with the peritonæm and pleura. Occasionally they ulcerate and the *echinococcus* dying atrophies, terminating in a spontaneous cure.

*Diagnosis.*—The diagnosis is frequently difficult. If a tumor is found giving dullness on percussion, with perhaps the hydatid fremitus, and under the microscope the aspirated liquid shows hooks and other parts of the cyst, a diagnosis would be reasonably certain. The prognosis depends upon the situation, size and rapidity of growth of the tumor.

*Treatment.*—Besides a spontaneous cure referred to above, the cyst may rupture, discharging its contents into the bowels, stomach, lungs, ureters, etc., completing a cure. But the treatment proper may be either medical or surgical. Arranged under the

---

*The head of the tibia is a common resting place for them.

first head we find recommended the administration of bromide and chlorate of potassium, chloride of sodium, male fern, oil of turpentine, calomel, kameela, etc. As a rule this method of treatment has been a failure, while the surgical treatment, viz., excission wherever practicable, and in other cases aspirating the cyst causing the death of the parasite, followed by atrophy of it, is the most satisfactory. Electro-puncture has been employed by an Icelandic surgeon with a success warranting further trial. Prophylactic measures should be employed where and whenever the disease is prevailing, such as destroying the excreta of the dogs from which the ova is indirectly introduced into the alimentary canal of man.

*T. nana.*—As regards the dwarf tape worm, unless Spooner's case* be genuine, there is but one solitary instance on record of its occurrence in the human body; moreover we have no evidence of its having existed in any other host. It was discovered by Dr. Bilharz, of Cairo, at the post mortem examination of a boy who died from inflammation of the cerebral membrane. Prodigous numbers existed. The largest specimen measured only one inch in length. To the naked eye these worms resemble short threads, and consequently they might very readily be overlooked. The head is broad and furnished with a formidable rostellum armed with a crown of hooks. The hooks have large anterior root processes, which extending unusually forward impart to the individual hooks a bifid character.†

*T. flavo maculata.*—This cestode was first discharged from an infant nineteen months old. The length varies from eight to twelve inches; the joints being very broad, narrowed from above downwards; near the middle of each joint may be seen a number of yellow spots, representing the male organs of generation, the outlets occurring all along one side of the body.

*T. lophosoma.*—This cestode measures about eight feet in length; breadth one-fifth inch, and one-thirteenth of an inch thick. There is a ridge running the entire length of the parasite, hence, the proposed common name "ridged tape-worm." The

---

* American Journal Medical Science, 1873.

† Cobbold.

reproductive papillæ are very prominent. The eggs have a diameter of about one eight hundredth of an inch.

For symptoms and treatment of the last three parasites, see *T. solium.*

BOTHRIOCEPHALIDŒ.—This family is charcterized by having two narrow lateral furrows without hooks on the head. Three species have been found inhabiting the human body.

*B. latus.*—This cestode derives its name from the width and shape of its articulations, being considerably broader than long. It is found in the cavity of the small intestines. The color is blueish-white, sometimes brownish in the middle. Length, very great, amounting in some cases, to over 2,000 articulations. Width, about one inch. The head is oval, one twenty-fifth of an inch in diameter, and capable of assuming various forms. The male and female organs of generation are distinct, those of the former being farther in front. The mature eggs are oval, $\frac{1}{370}$ of an inch long, provided with a lid for the escape of the embryo. The outer membrane of the embryo is covered with ciliæ, while within is a vesicle provided with three pairs of spiculæ, like those of the *Tœniœ.* The mature articulations are discharged from the bowels in chains after having evacuated the ova.

The history of the development of *B. latus* has not been determined with any degree of certainty, but as far as investigations have been carried, they point to the different varieties of fishes as the breeding places for it.

*B. cordatus.*—This cestode infests only the residents of Greenland, and corresponding longitudes. It is usually about one foot long, closely resembling the above as to its anatomy, except the head which is heart-shaped, the apex directed forwards.

The symptoms and treatment do not differ materially from those caused by other tape-worms.

*B. cristatus.*—I can find but two cases reported of this parasite (*Davaine). He states that it is nine or ten feet long, and is characterized by two prominences, together forming a crest covered by papillæ. The segments are one-half an inch in breadth, the entire worm being narrower than *B. latus.*

---

*Traite, art. Les Cestodes. Bibl. No. 2, p. 580.

* TREMATODES.—An order of *Platyhelminthes* not articulated, and the integuments free from ciliæ in the embryo. They are provided with one or more suckers, by which they attach themselves to the animal unwillingly acting as their host. The alimentary canal is forked, without an anus; the vascular system is undeveloped, but there is an urinary apparatus, with an excretory tube. They are mostly hermaphrodite; but as there is no connection between the organs of generation, it requires the union of two individuals for impregnation.

*Trematodes* exist in two forms, viz.: encysted and free. Their mode of development is very interesting. The perfect worm deposits an egg, which hatches into a sac, formerly known as *Opalina*, itself an entozoa, and once believed to be an Infusoria. This gives birth by internal germination to one or more *cercariæ*, or oval tailed organisms, which swim actively in the water, and if possible, enter the bodies of fishes, etc., and, either directly or indirectly, into the stomach of man. Here they develope into *Trematode* worms or liver flukes. We find them devided into several families, but one being interesting to us, viz.: Distomidæ, members of which we will proceed to describe.

*D. hepaticum.*—This has been found under the skin in various parts of the body, but it is most frequently found in the gall ducts and sac, where as many as 30 or 40 have been found in one patient.

The sexually mature fluke measures $\frac{3}{4}$ of an inch in length, and is about $\frac{1}{2}$ an inch through its greatest diameter. The body is very flat, with the anterior end so constructed as to form the head and neck. The mouth is found at the root of the neck; the reproductive organs in the mesial line just below. These organs are marked by dark spots and lines showing the extent of the male and female organs. The surface of the body is covered with minute spines which have diminished in size toward the posterior extremity.

*D. crassum.*—This fluke is about two inches long, and $\frac{5}{8}$ of an inch broad. The alimentary canal in this species is not branched, but double; the body pointed in front and rounded behind. But few cases have been recorded.

* A hole.

*D. lanceolatum.*—Three cases of the occurrence of this parasite have been recorded.

This is the lance-shaped fluke, and measures $\frac{1}{3}$ of an inch in length, and $1\frac{1}{2}$ lines in width; both ends are pointed, but the posterior is more obtusely so. The surface is smooth, without spines.

These are found in the biliary passages, usually existing without causing any untoward symptoms; but they occasionally are the cause of grave disorders, as the "dysentery of the Nile," etc.

The patient will have the symptoms of intestinal irritation; foul tongue; surface cold; pulse accelerated; indigestion; nausea; headache and diarrhœa; occasionally vomiting, and passing per ani blood persistently. The treatment would depend upon the symptoms; but expulsion of the parasite should be secured. Ordinary vermifuges are, as a rule, inefficient, remedies that will profoundly affect the system being indicated.

Great care should be exercised when eating oysters, clams, fish, etc., to see that the flesh is well cooked, as it is through them that the parasite is introduced into the body.

*D. hœmatobium* is found in the abdominal venous system, especially in the vena porta, and may occasion serious nervous and other disorders. It occurs in $33\frac{1}{3}$ per cent. of the population of Egypt. The males and females are distinct, the male being $\frac{1}{2}$ an inch long, and cylindrical; the female being filiform, and $\frac{4}{5}$ of an inch long. The surface of both sexes is smooth.

*D. heterophys.*—This minute parasite is $\frac{3}{4}$ lines in length, and was first found in the intestines. It is pear-shaped, the small end being anterior, while the entire surface is covered with spines. The ventral surface is provided with a peculiar disk, supposed to aid as a "hold fast" during the sexual act.

*Tetrastoma renale* is five lines long; found in the urine.

*Polystoma pinguicola* is eight lines in length; was found in a tumor connected with the ovary.

*P. venarum* is three lines in length; it has been found in venous blood and sputa of patients troubled with hæmoptysis. This species is doubtful, and is supposed by some to be the young of *D. lanceolatum.*

*D. ophthalmobium* is about 1-24 inch. in length, and of an oval shape. It was found in the eye of a child suffering from congenital cataract; frequently called "the eye worm." This species will probably prove to be the young of some of the above parasites.

The treatment is simply palliative when dealing with the parasites noticed above.

NEMATELMINTHES.—The *Nematelminthes* next claim our attention. They have cylindrical bodies, and are distinctly unisexual; there are no organs of special sense, but the alimentary canal is perfect throughout. But one order has been found in man, viz.:

* NEMATODES.—In these parasites the body tapers toward each end, and has a distinct muscular system; the mouth is at one extremity; the anus at, or near, the other; the alimentary canal consists of a thick-walled pharynx and the intestine, a simple tube representing the stomach and œsophagus; the nervous system consists of a ring of fibers and nerve cells around the pharynx; the sexual organs, as a rule, are distinct, those of the male being near the posterior extremity, while those of the female are near the center of the body. The spermatozoa are cell-like and amœboid.

About a dozen species are known to inhabit man, and may conveniently be arranged into four families, viz., ASCARIDÆ, STRONGLYLIDÆ, TRICOCEPHALIDÆ, and FILARIDÆ.

The ASCARIDÆ have two representatives.

*A lumbricoides*, inhabiting the intestine. The body is round, with a smooth surface, yellowish in color, tapering toward the head. The head commences abruptly by three tubercles which surround the mouth; the body is marked with transverse lines, and with four lines from the head to the tail. The males are about six inches in length, while the females are a foot, and even eighteen inches. The natural history of this species is as yet not fully known.

*Symptoms.*—The discharge of the worms is the most important and only reliable symptom. Many others are mentioned by writers, as itching of the nose, convulsions, grinding the teeth

* Threadlike.

while asleep, etc.—symptoms showing intestinal irritation. Sometimes the worms are vomited. A child living here was troubled with this parasite recently, and getting frightened at the appearance of the stranger after handling it, rubbed his eyes while crying, whereupon the lids became enormously swollen, completely closing them. Tr. of lobelia will reduce the irritation and swelling in a short time if applied to the parts.

*Treatment*:—The indications are, first to expel the worms present, and second to prevent their re-accumulation. The most efficient means of fulfilling the first is the administration of santonin, spigelia and senna, tansy, or wormwood.

They usually find their way into the system in the drinking water, hence avoid shallow wells, pools and creeks for water supply.

*A. alata*.—This is comparatively unimportant to the physician, being rarely found in the human subject. It is found in the domestic cat frequently.

For symptoms and treatment, see *A. lumbricoides*.

*A* (Oxyuris) *vermicularis*.—This is by far the most freqent of all the parasites infesting the human body. They are most frequently found in children. I have seen several cases occurring in the adult; one young lady aged 18, was troubled to the extent of preventing her from aiding in the housework owing to the intolerable itching and nervousness. The seat, or pin-worm, is of a fusiform shape, the male being about one-sixth, and the female one-third to one-half an inch in length. The female possesses a long, slender tail, terminating in three points, which are to aid the parasite in attaching herself to the intestine. The surface is white and transversely lined. They inhabit the rectum usually, but occasionally find their way into the urethra and vagina.

*Symptoms*.—This parasite usually causes an intense itching pain in the rectum, tenesmus, excitation of the genitals, etc. The pruritis ani is caused by an herpetic eruption there which, when present, should always excite suspicion. The irritation may occasion loss of sleep. and thus impair the general health. They are sometimes voided in large masses, agglutinated together with mucous.

*Treatment.*—The same anthelmintics used for *A. lumbricoides* may be used here; injection of salt and water once or twice daily are good; also of vinegar, camphor, etc. Santonin suppositories are usually effective, and are considered the best remedy by many.

In all cases where worms are known to exist in the bowels, attention should be paid to the general condition of the digestive organs.

STRONGYLIDÆ.—The family characteristics are: the body is round and sometimes very much elongated; the mouth is round, oval or triaugular, and located at the extreme end of the body; the tail of the male is furnished with a bursa emitting two spiculæ.

*S. gigas*—Is the largest nematode parasite known to infest man, the male measuring from ten inches to a foot in length and one-fourth inch. wide, while the female is over three feet in length, its transverse diameter being one-half inch. The body is of a reddish color, the head obtuse and furnished with a simple oval aperture surrounded by six chitinous nodules.

The mode of reproduction is vivparious. This worm is found in the kidneys, bladder, abdominal cavity and omentum in man, and in the liver and lungs in some of the lower animals. When found in the kidney the substance of that organ is disorganized and its place occupied with a sanguino-purulent liquid in a short time. It is rare in man.

*S. quadridentatus* (Dub.): Is found in the small intestines, and causes a form of anæmia by piercing the walls of the intestine producing hæmorrhage, and at the same time abstracting large quantities of blood for their own subsistance. It is frequently found in Europe, Africa and South America (Brazil), but rarely in North America.

This emotode is from three-eighths to one-half an inch long. The head is pointed and bent so that the mouth is toward the vertex. The body terminates in a sharply pointed tail in the female, and a blunt point in the male.

*Symptons.*—Resembling ordinary anæmia.

*Treatment.*—Turpentine is said to rid the person of this parasite by killing it.

*S. bronchialis.*—This was found in the bronchial glands of an emaciated subject. It is of the ordinary shape, of a yellowish color, the male being one-half, while the female about one inch in length, about one-fortieth inch broad. The bursa is nearly bell-shaped in this species.

TRICHOCEPALIDAE.—But two species of this family have been found in the human body, and one of them is one most dangerous of all parasites.

*T. dispar*, so called from the hair-like appearance of the cephalic extremity. It is nearly two inches in length, the capillary portion forming about two-thirds of its length. It has been found in the cæcum, colon, and rarely in the small intestine. It is propagated by means of ova, which may be found in the dejection with the aid of the microscope, a fact which might be utilized.

The *T. dispar* is found in bodies of persons who have died from various diseases, viz., typhoid fever (A. Flint), in one case enormous quantities being found in the cæcum, the patient having died from symptoms of meningitis. No lesions of that disease were found after death. J. L. Smith says he knows of no symptoms caused by its presence, and requires no treatment, although any of the parasiticides may be used, followed with a cathartic.

*Trichina spiralis.*—As stated above, this is one of the most dangerous parasites infesting the human body. Also found in the muscular tissue of the hog, rat, etc. As commonly found, it is in a quiescent, encysted state, occupying the voluntary muscular system. The cyst is oval, about one-tenth of an inch long, and when recent, quite colorless ; but after a time they become partly opaque, from a deposit of calcareous matter. Then they may be seen as minute, whitish specks scattered throughout the infected part.

This worm lies in the cavity of the cyst coiled upon itself hence its name. When stretched out it is cylindrical, $\frac{1}{28}$ of an inch in length and $\frac{1}{650}$ of an inch in diameter at its posterior extremity which is blunt and rounded, while the anterior is tapering and slender. The mouth and anus are located at either extremity, the alimentary canal extending in a direct line from one to the other. The sexes are separate, but the organs of generation are incompletely developed. The worm may be seen to be more sluggishly. In this imperfect and practically sexless condition the parasite may continue for an indefinite period of time retaining its vitality, but undergoing no perceptible change. When, however, a piece of flesh containing the cyst is eaten by man in a partially cooked condition, the flesh with the cyst wall is digested and the worms set free in the cavity of the intestine. They increase in size in two days, being developed into the perfect trichinæ. Fecundation now takes place by a union of the sexes, and in seven days from the introduction of the food the female discharges living embryos from the vulva in immense numbers (500 to 600), and as the generative act continues for seven to ten days, they probably give birth to at least one or two thousand living young. The embryos are now about $\frac{1}{200}$ of an inch long, resembling the mature parasite. They immediately penetrate the walls of the intestines and disperse over the body, being found in the intestinal walls, mesentery, diaphragm, peritonæum and pleura and in all of the *voluntary muscles, the passage being made very rapidly having been found on the eighth day after eating infested meat. They are quite free when first found in the muscular tissue, but soon become encysted and then grow to the proper size, there to remain quiescent until taken into the stomach of another animal when they pass through the same metamorphosis as did their parents.

This process of development of the adult trichinæ in the intestines and the dispersion of the young produces a very dangerous and sometimes fatal illness in the person called trichinosis. Its earliest symptoms are those of intestinal irritation, viz., abdominal pains, nausea, vomiting and diarrhœa, accompanied by anorexia and fever, followed by ædematous swellings of the face, body and limbs, with muscular pains and tenderness, especially on motion; the patient lies helpless with the extremities in a semiflexed position, any movement causing intense suffering. There is also difficulty of mastication and deglutition caused by the invasion of the corresponding muscles by trichinæ; hoarse-

*A case was reported for the Chicago *Tribune* during the winter of 1881-2, in which the *heart* was penetrated by trichinæ. Drs. N. S. Davis and R. L. Rea were in attendance.

ness and loss of voice by their presence in the larynx, and if in the diaphragm and intercostal muscles greatly impede respiration. In one case reported it was estimated that there were 30,000 to the cubic inch in the gastrocnemius, 70,000 in the biceps, and in the deltoid over 90,000. "The severity of the disease depends on the quantity of trichinous food ingested, and the number of living parasites it contained." In severe cases death may ensue within two days from the intensity of the diarrhœa and fever, but usually in does not occur until the fourth or fifth week. If the patient lives after that period, he stands a fair chance of recovering, gradually returning to comparative health, but suffering from some stiffness of the muscles. The trichinæ are then encysted and remain so. It is doubtful if they are ever absorbed, having been found in the flesh of a man eighteen years after the attack of the disease.

*Treatment.*—The indications for treatment are, first, to drive the parasite from the alimentary canal (per ani), and second, if possible, destroy it. Cathartics have been given to fulfill the first indication, while benzine has been employed to kill the parasite, with some success. Carbolic acid has been recommended. The fever and pain are to be controlled by appropriate treatment. These remedies are to be used while the trichiniæ are yet in the intestines. Lately Prof. B. Howard Rand has proposed a mode of treatment to be tried after they have entered the tissues. He proposed to kill them by mercurial salivation, getting the patient completely under the influence of mercury, which being a parasiticide he thinks will destroy the trichinæ. If mercurials are for any reason contra-indicated, he recommends sulphur to be used in the same manner. Prevention is the true way of meeting the difficulty, and as the parasite is introduced into man by means of pork, all should see that that kind of meat is heated at least above 160° F., as that is sufficiently hot to destroy the cyst.

FILARIDÆ.—This family consists of several members having hair-like forms, and nearly all are parasitic.

*F. medinensis.*—This parasite is from 6 inches to 4 feet in length and one-ninth of an inch in a diameter. It prevails in Africa, India, and Arabia in Asia, and the United States.* The body

* This parasite occurred in the person of a relative residing in Massachusetts.

is cylindrical ending in a tail. The head is convexly flat with the mouth in the center, and that organ surrounded by four papillæ. They are viviparous. The male is unknown. The larvæ pass a portion of their existence within the bodies of some crustaceans. They then, in some manner, as yet unknown, pass into the human body probably by means of the drinking water and reach maturity. The female gets beneath the skin of a human being developing with its contained young into a whip-like cord just beneath the integument. To reach maturity requires a variable length of time, probably less than one year. It then approaches the surface causing a small ulcer through which it discharges the young after which it may be drawn out. The parasite usually causes no inconvenience; but occasionally it causes severe pain and even death. The Egyptians pour cold water on the skin over the parasite sometimes causing it to crawl out.

Tr. assafœtida in 30 drop doses three times each day will poison the worm.

*Preventive.*—Persons living in infected regions should filter the drinking water.

*F. popillosa* (*F. oculi*, of veterinary writers) is commonly found in the eyeball, also in the tissues, thorax, abdomen, membranes of the brain, muscles and cellular tissue. The males and females are three and seven inches respectively. The head is broad with a wide hard mouth armed with two denticles; two other denticles will be found on the sides of the neck and sixteen on the tail—eight on a side.

The worm migrates into different tissues, the track, of a yellowish color being readily seen by the naked eye. The natural history is as yet unknown. When occurring in the eye of the horse they are frequently extracted by veterinary surgeons.

*F. lentis* measures from one-fifteenth to three-fourths of an inch long, found in the crystaline lens. Probably this is the sexually immature *F. lacrymalis* of Gurlt.

*F. trachelis* measures about one-fiftieth inch long; it was found in the trachea and larynx of a person dying from some disease of the lower extremities.

*F. bronchiaeis* is from one-half to one and one-half inches in length, and found in the bronchial glands. The tail of the male has a bursa bilobed and and bell-shaped. The tail of the female is pointed. They are of a pale yellow color and viviparous.

*F. sanguinis hominis*, Lewis, is the immature, *F. bancrofti* Cobbold. The mature female is about the size of a hair and three inches in length. The mouth is circular and destitute of papillæ. The neck is narrower than the body with the reproduction outlet near the head; the tail is blunt, the anus near the tip. The immature form as existing in the blood, is $\frac{1}{75}$ inch long. The parasite always exists in the vascular system rarely causing much inconvenience; but in the African race they occasionally cause grave disorders, such as chyluria and elephantiasis, and derangements of the nervous system. They have been found in abscesses of the lymphatic glands. They seem to be conveyed into the human blood by means of the musquito, having been found in the stomach of that insect.

INFUSORIA.—Is the highest division of Protozoa; they possess a mouth, a rudimentary digestive cavity and vibratil ciliæ. They are extremely minute and have the power of swimming about freely; but some species are fixed in the adult state.

*Paramecium coli* has frequently been seen in the dejections of fever patients, and those suffering from Cochin-China diarrhœa.

*Cercomonas urinarius*, has been found in the bladder and urine of a patient by Hassel.

*C. soltans* was found in the ovaries by Ehrenburg.

*C. hominis*, found in the colon and evacuations of cholera patients.

*Tricomonas vaginalis*, found in vaginal mucous.

### PART II.

ECTOZOA.—These are animals which live upon the body of other animals, in some cases within the integument. They are usually articulates, and belong to a very low order of INSECTA and *arachnoida*. It has been a question and but recently satisfactorily answered, if these creatures were the cause of certain diseases, or the diseased condition the proper soil for their progagation. We are indebted to Schiodte, of Denmark, for the settlement of this.

The DIPTERA is the only order of insects represented among human ectozoa. It is represented by the family PEDICULIDÆ, consisting of three species.

*P. capitis*, or common head louse, is a small wingless animal, with a small thorax and large abdomen. The head is provided with hooks and beak to draw the blood from its victim, after attaching itelf by means of the beak. There it revels in human gore, to the intense misery of its unfortunate host. The eggs are oval, and attached to the hairs.

*Treatment.*—First enforce cleanliness; then an ointment prepared from sulphur, mercury, seeds of sabidilla and stavesacre, root of pyrethrum; also, any of the essential oils and alcohol may be applied over the parts infested. The eggs or "nits" may be removed by frequent washings with alcohol or vinegar.

*P. vestimenti*, the body louse, closely resembles the *P. capitis.* The eggs are round, yellowish white, and are always found in the folds and seams of the infested clothing—the home of the parents.

*Treatment.*—The proper way to destroy both insects and eggs is to place the clothing in the oven, and subject them to a considerable baking; then enforce cleanliness.

*P. pubis*, or crab louse, is found where the coarse hair grows, viz., the pubis, legs, chest, axillæ, beard, eyebrows, etc., but never enters the hair. Each variety has its own place of habitation, live there and there only, never trespassing upon the soil of its neighbors.

*Treatment.*—See *P. capitis.*

*Œstrus humanis* is a bot fly, which deposits its eggs in the skin of man, probably in the sweat tubes, where they hatch into larvæ or "grubs." They form boils, or even ulcers.

*Treatment.*—The opening in which the *œstrus* introduced the eggs should be enlarged, and the grub pressed out.

(I have met with one case, occurring in the person of my grandmother.)

PULICIDÆ is the flea family. It is represented by two species.

*P. irritans*, or common flea, is occasionally seen on the human body. The bite is very irritating, however usually causing no serious symptoms.

*Treatment.*—Cleanliness and insecticides.

*P. penetrans*, or chigœ, is smaller than the *P. irritans*, with a proboscis as long as the body. The female only penetrates the skin, where she swells into a white globular vesicle about the size of a pea within a few days, causing intense pain.

*Prognosis and treatment.*—The parasite should be withdrawn with a needle, the entrance being easily seen, marked by a little red spot. When neglected, or carelessly ruptured, the young are scattered in the wound, where they mine passages, causing very serious gangrenous ulcers and sometimes death. Amputation would then be indicated.

ARACHNIDA.—Is represented by the order ACARINA, containing the mites. One family of this order will be treated of, viz., ACARIDA, which is divided into several sub-families, two of which are interesting to us—*Hypoderinæ* or subcutaneous mites, and *Sarcoptinæ*, or itch mites.

*Arachnida* are distinguished by the following characteristics: Head and thorax amalgamated into one piece; no true antennæ; four pairs of legs; breathing various. Contains spiders, ticks, scorpions, etc.

*A. folliculorum*, or pimple mite. These are harmless parasites, found in the ducts of sweat glands, especially those about the nose. Length, 1-100 inch. It has been asserted that no one is free from them. When present in large numbers they may excite irritation of the skin, producing an acne-like eruption. I have in my possession two specimens of this parasite, taken from the skin of a dog*. In this case there was an extensive eruption wherever the pimple mite existed.

*Treatment.*—If any is necessary, any of the parasiticides mentioned for *P. capitis* may be applied.

*A. scabies*, or itch mite, the cause of itch. It is a minute whitish insect, from 1-100 to 1-50 inch long, the male being least often found. The female is usually found at the end of the burrows they mine where she deposits her eggs. She makes her

* By Prof. Lester Curtis.

way beneath the epidermis by means of suckers or disks upon the legs, and bristles on the back directed backward. Hahneman thought the itch was the primary cause of all diseases. Years ago there were several varieties of itch, viz., "Jackson's itch," "Seven year itch," "Army itch," etc., but they are now known to be caused by the presence of the same insect, *A. scabei.* When burrowing in the skin the mite causes intense itching, and sometimes a vesicular eruption. If the disease progresses beyond this stage, it is due to the scratching done by the patient. As the mite requires a soft tissue to work in, they are usually found between the fingers.

*Treatment.*—The disease will remain until the parasite is destroyed. Cause the death of the mite, and then reduce the inflammation. First cleanse the parts thoroughly, and soften with soap and hot water; then apply common sulphur ointment, carbolic acid, kerosene, etc. Treat the vesication and inflammation with cosmoline carbolized.

---

